# Stroke incidence in Indigenous, minority populations: a review of methods for studying stroke in Aboriginal and Torres Strait Islander Australians

**DOI:** 10.3389/fstro.2023.1270136

**Published:** 2023-11-01

**Authors:** Anna H. Balabanski, Lee Nedkoff, Angela Dos Santos, Alex Brown, Timothy J. Kleinig, Amanda G. Thrift, Judith M. Katzenellenbogen

**Affiliations:** ^1^Department of Neurosciences, Central Clinical School at Monash Health, Monash University, Melbourne, VIC, Australia; ^2^Department of Medicine and Neurology, Melbourne Brain Centre at the Royal Melbourne Hospital, Melbourne, VIC, Australia; ^3^Department of Stroke Medicine, Alfred Brain, Alfred Health, Melbourne, VIC, Australia; ^4^School of Population and Global Health, Cardiovascular Epidemiology Research Centre, The University of Western Australia, Perth, WA, Australia; ^5^South West Sydney Clinical School, University of New South Wales, Liverpool, NSW, Australia; ^6^National Centre for Indigenous Genomics, Telethon Kids Institute, The Australian National University, Canberra, ACT, Australia; ^7^Department of Neurology, Royal Adelaide Hospital, Adelaide, SA, Australia; ^8^Department of Medicine, School of Clinical Sciences at Monash Health, Monash University, Melbourne, VIC, Australia

**Keywords:** stroke, Indigenous, minority, methodology, incidence, populations

## Abstract

Declining worldwide or national stroke incidence rates are not always mirrored in disadvantaged, minority populations. Logistical barriers exist for effective measurement of incidence in minority populations; such data are required to identify targets for culturally appropriate interventions. In this comparative review, we aimed to examine whether “gold-standard” methodologies of stroke incidence studies are most effective for minority populations. We compared three studies of stroke incidence in Aboriginal Australians, each using different methodologies of case ascertainment. In Study 1, “gold-standard” population-based methods were used, while in Study 2, a retrospective hospital-based cohort design was utilized, and in Study 3, whole-of-population linked hospital and mortality data was employed. Study 1 captured both in-hospital and out-of-hospital stroke events but had a small sample size for Aboriginal patients. Study 2 provided a larger sample size while still allowing for clinical and radiological subtyping of stroke but was subject to selection bias and was limited to hospitalized cases. Study 3 had a large sample size and allowed for subgroup analysis, though lacked clinical adjudication and had large proportions of ‘undetermined stroke'. Despite diagnostic imprecision, we recommend a paradigm shift in measuring stroke incidence in Indigenous, minority populations, favoring the use of whole-of-population data linkage including non-hospitalized stroke deaths, over resource-intensive prospective methods, where more suitable for the target population.

## Introduction

The Global Burden of Disease study shows declining stroke rates worldwide (Stark et al., 2021). However, these improvements are not always reflected in disadvantaged, minority populations (Trimble and Morgenstern, 2008; Feigin et al., 2015; Balabanski et al., 2018). If improvements are not at least commensurate, stroke disparities for such minority populations will widen, underscoring the need to monitor stroke rates in minority groups. Many minority Indigenous populations have greater rates of stroke than their respective non-Indigenous populations, though high-quality, contemporary data are sparse (Zhang et al., 2008; Atzema et al., 2015; Feigin et al., 2015; Balabanski et al., 2018; Siri et al., 2020). Despite distinctive cultures, languages, and epistemologies, most Indigenous minority populations experience poorer socioeconomic and health outcomes, in part due to the ongoing impact of colonization.

Aboriginal and Torres Strait Islander (hereafter, respectfully referred to as Aboriginal) Australians comprise 3.2% of the Australian population (Australian Bureau of Statistics, 2021a), inclusive of diverse peoples, communities, and language groups. Aboriginal Australians represent the world's oldest continuous living culture, and their strength, resilience, and continuing connection to the land should be celebrated (Commonwealth of Australia, 2018). The 2007 United Nations Declaration on the Rights of Indigenous Peoples recognized their right to self-determination (United Nations, 2007). However, the ongoing effect of colonization on Aboriginal Australians has directly impacted individuals' and communities' physical, social, emotional, cultural, and spiritual wellbeing. Consequently, Aboriginal Peoples typically have poorer health outcomes than non-Aboriginal Australians, underpinned by the social determinants of health, including racism and discrimination, impoverished living conditions, limited education opportunities, increasing rates of imprisonment, and a greater mental health disease burden. Additionally, there are numerous barriers to accessing health services at the individual, provider, health system and policy level, each exacerbated by cultural and language barriers common to many Indigenous and minority groups (Australian Institute of Health Welfare, 2020; Levine et al., 2020; Office of the National Rural Health Commissioner, 2022).

Health research has often failed to improve Indigenous health outcomes, with methods often unsuitable and action not always taken (Bainbridge et al., 2015; National Health Medical Research Council, 2018). For stroke, barriers exist to effectively measure incidence in minority populations, many of whom might be geographically dispersed and consequently have limited access to tertiary healthcare. Such is the case among Aboriginal Australians.

In this comparative review, we aimed to examine whether “gold**-**standard” stroke incidence study methods are appropriate for Indigenous, minority populations. We compared stroke incidence in three studies from the South Australia and Northern Territory Stroke (SAiNTS) Study of stroke epidemiology in Aboriginal Australians (Balabanski et al., 2018, 2020, 2023). All three studies were designed and undertaken in response to community priorities with Indigenous stakeholder and researcher oversight and governance to ensure the research was done for the benefit of Aboriginal Australians.

## Methods

Stroke incidence studies require a population-based design, using a known source population (denominator) defined at a national, regional, or sub-regional level. Within this population, cases of incident (first-ever-in-a-lifetime) stroke over a specified period (numerator) can be identified retrospectively or prospectively in this population-based approach.

The “gold-standard” methods for stroke incidence studies, as first outlined by *Sudlow and Warlow*, involve “*complete, community-based case ascertainment, based on multiple overlapping sources”* (Sudlow and Warlow, 1996) (including fatal and non-fatal non-admitted cases) to identify incident stroke events prospectively in a well-defined, stable population (Sudlow and Warlow, 1996). Accompanied by standardized methods for reporting, the criteria provide a framework for interstudy comparison, although they have adapted as treatments and definitions evolved (Feigin et al., 2018). The methodology is both time- and resource-intensive, often feasible only in regions with advanced infrastructure and large, stable populations.

Retrospective case ascertainment offers an alternative approach. Clinical and radiological data can be used for case identification, validation, and analysis. Alternatively, administrative (non-clinical) data sources can provide whole-of-jurisdiction or multijurisdictional case ascertainment. Unlinked administrative data (individual admission stroke-coded records, without the ability to person-link records) cannot accurately measure incidence, as numerators are not defined at the person-level such that incident and recurrent events cannot be distinguished. In contrast, the use of person-linked administrative data allows for the identification of incident stroke cases within the study period (Katzenellenbogen et al., 2010). A “lookback” period prior to the study enables the identification and exclusion of patients with prior stroke events (Katzenellenbogen et al., 2010).

The three studies described in this review were each conducted using different methodologies to obtain incident stroke numbers in Aboriginal Australians (numerators). The first study comprised “gold-standard” population-based methods to study stroke incidence in South Australia (Balabanski et al., 2023). The second involved a retrospective hospital-based study of stroke incidence and in-hospital deaths in Central Australia (Balabanski et al., 2020). The third incorporated use of whole-of-population linked hospital and mortality data from South Australia, the Northern Territory and Western Australia (Balabanski et al., 2023). In all instances census population estimates were used as denominators.

### Indigenous identification

In Australia, Indigenous status is determined by self-identification, descent, and community recognition (Christensen et al., 2014). In the first and second studies, Indigenous patients were identified when individuals within the study had self-identified as Aboriginal. Within administrative data sources included in the third study, Indigenous status was determined using an algorithm using self-reported Indigenous identification as recorded in the separate administrative datasets.

### Statistics

We calculated incidence rates as the overall number of first-ever stroke events divided by the total number of people in the population (crude rates), and standardized to the World Health Organization world population (age standardized rates) (Ahmad et al., 2001; Armitage et al., 2008).

## Results

We identified several advantages and disadvantages of each of the methodologies used to ascertain cases to determine the incidence of stroke in Aboriginal Australians in the SAiNTS studies (Table 1). These studies also collectively incorporated a unique approach for analyzing and reporting the age-standardized incidence of stroke in this minority population, by providing age-standardized rates of both the total population and in two broad age groups, dichotomised at age 55 years.

**Table 1:** Advantages and disadvantages of different methodologies in population-based epidemiological studies of stroke incidence in minority populations.

**Methodology**	**Summary of study**	**Strengths**	**Limitations**
**Study 1** Prospective “gold-standard” population-based study	Pooled results of two population-based studies of patients with stroke in urban (2009–2010) and rural (2009–2011) South Australia Aboriginal patients *n =* 13 Aboriginal population denominator *n =* 13,225	• Gold standard methodology, providing the highest quality evidence for the facilitation of healthcare planning, priority setting and resource allocation	• Time and resource intensive • Limited to certain regions • Potentially small sample size, limiting precision and subgroup analysis • May miss events in mobile populations
**Study 2** Retrospective hospital-based cohort study	Retrospective case analysis of all patients admitted to hospital with stroke in Central Australia (2011–2014) Aboriginal patients *n =* 74 Aboriginal population denominator *n =* 16,756	• Less resource intensive than “gold standard” methodology • Enables clinical and radiological subtyping of stroke	• Subject to selection bias (including ascertainment, healthcare access bias and missing information), information bias and confounding bias (Delgado-Rodríguez and Llorca, 2004) • Precision may be low if there are few stroke events in the hospital catchment • May miss events in mobile populations
**Study 3** Linked data analysis	Whole-of-population person-linked hospitalization and mortality data of patients with stroke in Western Australia, South Australia and the Northern Territory (2012–2015) Aboriginal patients *n =* 675 Aboriginal population denominator *n =* 118,224	• Large sample size, allowing for subgroup analysis and high precision • Allows for whole-of-jurisdiction or multijurisdictional analysis • May capture events in mobile populations • Cost-effective alternative to “gold standard” population-based studies • Ability to evaluate trends regularly	• Nonfatal events which are not hospitalized are not captured (likely < 1% of all events) (Katzenellenbogen et al., 2010) • Private hospital data for certain jurisdictions not captured (likely small proportion of all events) (Leyden et al., 2013) • Relies on administrative data without clinical correlation, particularly limiting evaluation of stroke types (especially for out-of-hospital fatal events) • Short lookback periods may not capture distant events, potentially overestimating incidence rates resulting from misclassifying incident/recurrent events.

### Comparison of case ascertainment

In the “gold-standard” study (Balabanski et al., 2018), we captured in-hospital and out-of-hospital stroke events, thus comprising a true estimate of stroke events and the full spectrum of stroke severity (Sudlow and Warlow, 1996). This approach also enabled diagnostic confirmation of cases, thereby distinguishing between strokes, transient ischaemic attacks, and mimics, and for evaluation of pathological stroke types and etiology (Sudlow and Warlow, 1996). However, using this method we only identified 13 cases of stroke in Aboriginal people, while 419 cases were identified in non-Aboriginal people. Consequently, confidence intervals were wide and detailed subgroup analysis was not possible.

In the second study (Balabanski et al., 2020), we identified stroke admissions at Alice Springs Hospital (in the Northern Territory) between 2011 and 2014 using established diagnosis codes from the International Classification of Diseases, 10th revision, Australian Modification (codes I60, I61, I62.9, I63 and I64) using all diagnosis fields (≤20). Case information from hospital records included clinical and radiological data, enabling retrospective adjudication of stroke type and etiology, as well as in-hospital deaths. This approach was far less resource intensive than “gold-standard” prospective methodology, with fewer personnel required to identify cases through administrative data sources with retrospective case evaluation. A more extended period for case ascertainment may improve case numbers for minority groups (i.e., Aboriginal people with stroke), as demonstrated in this study where 74 Aboriginal people with first-ever stroke were identified during the four-year study period, despite a similar population denominator of Aboriginal patients to that in the first study. Disadvantages of this study method included reliance on potentially inaccurate hospital coding to identify cases, and inability to identify non-hospitalized events or events within the defined population but out-of-catchment of the hospital. However, because all patients with suspected stroke in Central Australia are taken to Alice Springs Hospital, this lowers the likelihood that patients were out-of-catchment of the hospital, therefore allowing for a quasi-population-based design. Furthermore, retrospective diagnosis of stroke mimics, transient ischaemic attacks and stroke may be difficult to distinguish with certainty, depending on clinical accuracy at the time of medical record entry.

The third study (Balabanski et al., 2023), comprising whole-of-population, person-linked hospitalization and death registry data from three jurisdictions (home to ~20% of Australia's Aboriginal population) enabled capture of the largest number of Aboriginal people with stroke (*n* = 675), including fatal and non-fatal cases. This provided insights into stroke incidence by sex, jurisdiction, remoteness of residence and stroke type. Broad geographic coverage minimized the chance of missing events in a potentially mobile population. However, this approach was limited by the lack of clinical details leading to potential inaccuracies in demographic details and a lack of specificity in stroke type coding, especially for out-of-hospital fatal events. Additionally, as stroke events may have occurred in individuals prior to the lookback period, there is potential to overestimate incident events. Finally, non-hospitalized non-fatal events cannot be identified using this approach, although this likely represents a minority of stroke cases (<1%) in Australia (Katzenellenbogen et al., 2010).

### Methods for analyzing and reporting age-standardized incidence rates for stroke

**Figure 1:**
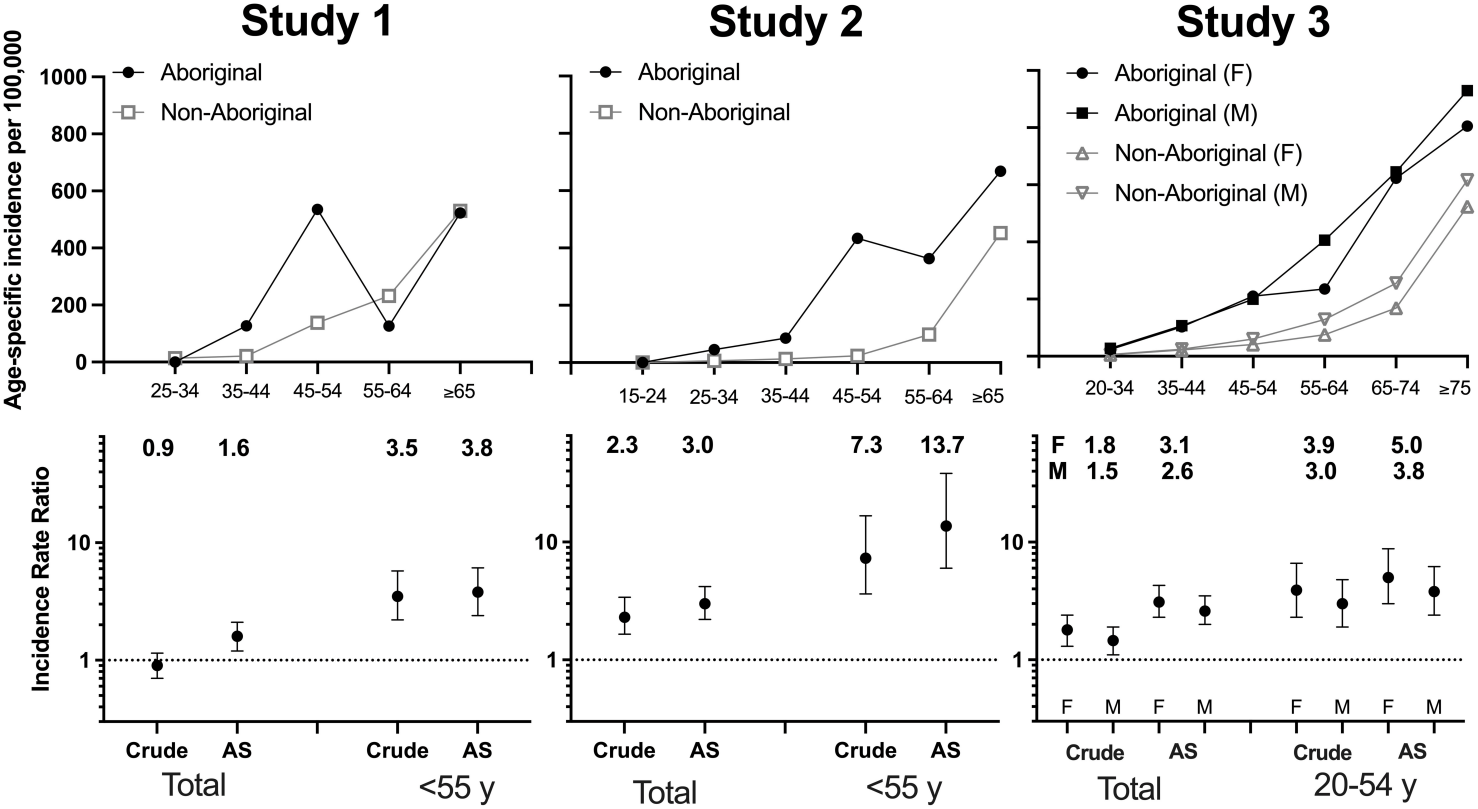
Age-specific incidence rates and crude and age-standardized rate ratios of stroke in Aboriginal and non-Aboriginal patients in the three SAiNTS Studies. AS, age-standardized.

In all three studies, incidence rates of stroke were greater in Aboriginal than in non-Aboriginal people. Disparities in age-specific rates between Aboriginal and non-Aboriginal Australians were relatively greater in the younger age-specific bands (Figure 1). These disparities reduced in successively increasing age bands, noting that low case numbers resulted in instability of rates in Study 1 and, to a lesser extent, Study 2. When standardized to the World Health Organization world population (Ahmad et al., 2001), age-standardized incidence rates in the Aboriginal population overall were greater than crude rates, whereas age-standardized rates were similar to or lower than crude rates in the non-Aboriginal population. Consequently, greater disparities in incidence between Aboriginal and non-Aboriginal Australians were observed using age-standardized rate ratios than crude rate ratios (Figure 1). When calculating age-standardized rate ratios in those aged <55 years, substantially greater disparities in stroke incidence were observed compared to age-standardized rate ratios for the overall cohorts (Figure 1). In Study 1 (*n* = 13 Aboriginal patients), the age-standardized incidence rate ratio of stroke for all adult ages was 1.7, notably lower than the Studies 2 (*n* = 74) and 3 (*n* = 675), where the rate ratios were ~3.

## Discussion

This body of work provides insights into measuring the burden of stroke in Aboriginal Australians, a minority population with incidence rates that differ substantially from those of the non-Indigenous Australian population. By comparing methodologies of case ascertainment and data analysis in the three studies, we provide important insights into conducting studies in minority populations where stroke incidence may differ substantially from that of the general population of a country or region.

We found that evaluation of age-standardized stroke incidence in Aboriginal Australians using “gold-standard” prospective ascertainment was limited and imprecise due to low numbers of cases ascertained. There was instability in trends in age-specific incidence, and evaluation of incidence by stroke type and other subgroups was not possible. In minority populations, subgroup analysis can only be performed when case ascertainment is sufficiently large, highlighting the advantages of using linked administrative data sources at the expense of clinical adjudication of stroke types. Furthermore, when examining overall incidence rates, crude rates did not represent the true burden of stroke in the Aboriginal population due to the younger age structure of the population. Finally, these studies collectively demonstrate the utility of determining age-standardized rates within two broad age bands, to highlight where the greatest degree of disparities between minority and non-minority populations may arise.

### Methods of case ascertainment

The most effective method for monitoring burden among Aboriginal and Torres Strait Islander peoples is via whole-of-population data linkage. In the Aboriginal Australian population, “gold-standard” prospective methods for measuring stroke incidence resulted in low case numbers with subsequent imprecise incidence estimations. These studies were performed (and are only feasible) in metropolitan and regional areas where Aboriginal populations were low, and would be difficult to perform in remote Australia, where health disparities may be greatest. Further, such studies are generally undertaken over relatively short periods. Therefore, it does not appear to be the most effective way of monitoring the burden of stroke in this population. Retrospective case analysis still allows for aetiological subtyping, while improving case volume compared with a prospective study design. Acknowledging the inevitable imprecision of using administrative data without clinical validation, linked data analysis of large administrative datasets containing multiple years of data is likely to be more suitable. This approach allows for larger cohorts, which provide more robust estimates of stroke incidence to help determine where prevention activities and care should be focused. Estimates will be further improved by greater accuracy of administrative data through improved stroke coding and optimized information systems datasets, including ensuring accuracy when recording Indigenous status.

Case ascertainment should include hospitalized and non-hospitalized strokes (i.e., from death records) when measuring incidence using administrative data. A recent Australian investigation into algorithms for identifying stroke in administrative data provided evidence that the exclusion of non-hospitalized stroke deaths from the algorithm resulted in a substantial underestimate in incident stroke counts, with the mortality contribution differing over time and for different sub-groups of the population (Youens et al., 2023). Given the large fatality from stroke among Aboriginal Australians, incidence rates should include these non-hospitalized stroke deaths in estimates to better evaluate the burden of stroke in this population.

However, studies utilizing administrative data are subject to a greater potential for bias due to inaccuracy in coding, when compared with “gold-standard” studies. As such, investigators must consider the potential impact of selection bias (including ascertainment and healthcare access bias), and confounding bias when interpreting results of studies not utilizing “gold-standard” methods (Delgado-Rodríguez and Llorca, 2004).

### Importance of accurately determining Indigenous status

In research, numerous barriers exist to identifying Indigenous individuals, creating potential for under-ascertainment (Thompson et al., 2012). In many countries, administrative systems do not routinely capture Indigenous status. Despite rigorous methodology to identify Indigenous individuals in all three studies, under-identification of Indigenous individuals may have occurred. In the first and second studies, accuracy may be limited by the individual's willingness to self-identify and accurate documentation and coding in the health system. In the third study, under-identification may have occurred, particularly among people living in metropolitan areas and areas of lower disadvantage (Thompson et al., 2012). Similarly, there is potential for errors in accuracy of Indigenous status in the population denominators, as evidenced by a change in the proportion of the Australian population identifying as Indigenous between Census years, not wholly accounted for by demographic factors (Australian Bureau of Statistics, 2021b).

Given the potential for under-identifying Aboriginal individuals, incidence rates presented in this body of work may underestimate the true rates within the Aboriginal population. Thus, disparities may be greater than those reported. Conversely, under-identification of Indigenous individuals in the Census data may result in an overestimation of rates. This illustrates the need for ongoing improvements in accuracy of documentation of Indigenous status within healthcare systems and Census data. Some countries with Indigenous populations, including Sweden and Canada, do not include direct Indigenous identification within administrative health data (Sjölander, 2011; Bougie, 2021). While the reasons for this differ between countries, this creates significant barriers to performing administrative data-based stroke incidence studies.

### Reporting age-standardized incidence

Age-standardization is critical when reporting incidence rates of stroke even within broad age bands. Crude rates are heavily influenced by the age-structure of the study population, thereby limiting comparison between different population groups (Belbasis and Bellou, 2018). Even if age-specific rates are consistently greater in a population with a younger age structure, crude incidence rate may still be less than for an older population. Thus, comparisons of crude rates can be very misleading (Armitage et al., 2008). This is particularly important for populations such as Aboriginal Australians, where the age-structure differs substantially from that of non-Aboriginal Australians (Australian Institute of Health Welfare, 2011). These studies collectively demonstrate limitations of calculating age-standardized rates that provide a single summary measure over all ages when evaluating the burden of stroke in the Aboriginal population, and perhaps other Indigenous and disadvantaged populations with high health burdens. Critically, this may obscure disparities in incidence in the population suffering the most substantial stroke-related societal impact, namely, stroke onset during peak years of raising a family and employability. Clinically, this is also important since making explicit the high risk in relatively young Aboriginal people will prompt investigation and treatment of stroke risk factors from earlier ages in Aboriginal patients and reduce missing early signs of stroke/TIA. Previous international incidence studies incorporating Indigenous populations have not incorporated age-standardized rates within broad age strata. Therefore, we recommended that future studies are conducted using such an approach and the authors consider reanalysing previous research using this methodology.

### Limitations

We describe research methods in the Aboriginal Australian population without examining studies in other Indigenous, minority populations. Consequently, our recommendations cannot be directly applied to studies in other regions. We encourage investigators to consider how the implications of our findings may be relevant to stroke incidence studies in different minority population groups.

An additional limitation relevant to these studies and to other epidemiological studies involving Indigenous, minority populations is accuracy of Indigenous identification.

### Recommendations

We recommend that, where “gold-standard” studies may be limited by feasibility in terms of ascertainment, high-quality linked administrative data may be used as an alternative for Indigenous or other minority populations, particularly where they can be identified in the data. This provides the opportunity to monitor stroke hospitalisations and deaths nationally to help identify targets for improved service delivery.

We also recommend that, where relevant, age standardization within stratified groups (i.e., younger and older age bands) should be undertaken to highlight the age groups in which disparities occur.

We recommend that these data include reliable documentation of Indigenous (or other minority) status and accurate data on stroke classification and comorbidity burden. Improved Indigenous/minority identification may be achieved through the inclusion of identifiers in administrative data sources, targeted staff education, optimizing documentation and coding within administrative systems, and continuous quality improvement activities. Importantly, tackling individual and systemic racism may encourage self-identification. In Australia, this could facilitate further linked data analyses which could be used to analyse stroke incidence by Indigenous Regions and evaluate trends in stroke incidence over time.

Importantly, any study undertaken with Indigenous or minority groups should also be designed and conducted with appropriate oversight and governance of the relevant population group, as outlined by Huria et al. (2019) in the CONSIDER statement, a “*collaborative synthesis and prioritization of national and international research statements and guidelines”* (Huria et al., 2019) and following CARE principles of Indigenous data sovereignty (Research Data Alliance International Indigenous Data Sovereignty Interest Group, 2019). Future research should be designed to inform the development of public and healthcare policy, and reform of health services. Priorities for health service reforms should be determined by the relevant communities and stakeholders, to reduce inequities and improve stroke outcomes in these populations.

## Conclusions

“Gold-standard” studies provide the best-quality data for determining rates and trends in stroke incidence in most populations. However, the feasibility of performing “gold-standard” studies, which must include a sufficiently large catchment and long duration for adequate ascertainment, may create a barrier to studying the incidence of stroke in Indigenous and other minority populations. We recommend that the approach be tailored to the individual population and conducted in consultation with relevant stakeholders.

## Author contributions

AHB: Conceptualization, Data curation, Formal analysis, Methodology, Project administration, Visualization, Writing—original draft, Writing—review and editing. LN: Formal analysis, Methodology, Writing—review and editing. AD: Supervision, Writing—review and editing. AB: Conceptualization, Funding acquisition, Supervision, Writing—review and editing. TK: Conceptualization, Formal analysis, Methodology, Writing—review and editing. AT: Conceptualization, Funding acquisition, Methodology, Supervision, Visualization, Writing—review and editing. JK: Conceptualization, Formal analysis, Methodology, Supervision, Visualization, Writing—original draft, Writing—review and editing.

## References

[B1] Ahmad O. B. Boschi-Pinto C. Lopez A. D. Murray C. J. Lozano R. Inoue M.. (2001). Age Standardization of Rates: A New WHO Standard. Geneva: World Health Organization.

[B2] Armitage P. Berry G. Matthews J. N. S. (2008). Statistical Methods in Medical Research. New York, NY: John Wiley and Sons.

[B3] Atzema C. L. Khan S. Lu H. Allard Y. E. Russell S. J. Gravelle M. R.. (2015). Cardiovascular disease rates, outcomes, and quality of care in Ontario métis: a population-based cohort study. PLoS ONE 10, e0121779. 10.1371/journal.pone.0121779 25793978 PMC4368556

[B4] Australian Bureau of Statistics (2021a). Aboriginal and Torres Strait Islander people: Census. Available online at: https://www.abs.gov.au/statistics/people/aboriginal-and-torres-strait-islander-peoples/aboriginal-and-torres-strait-islander-people-census/latest-release (accessed June 3, 2023).

[B5] Australian Bureau of Statistics (2021b). Estimates of Aboriginal and Torres Strait Islander Australians. Available online at: https://www.abs.gov.au/statistics/people/aboriginal-and-torres-strait-islander-peoples/estimates-aboriginal-and-torres-strait-islander-australians/latest-release#cite-window2 (accessed June 3, 2023).

[B6] Australian Institute of Health and Welfare (2011). Principles on the Use of Direct Age-Standardisation in Administrative Data Collections: For Measuring the gAp Between Indigenous and Non-Indigenous Australians. Canberra, ACT: AIHW.

[B7] Australian Institute of Health and Welfare (2020). Aboriginal and Torres Strait Islander Health Performance Framework 2020 Summary Report. Canberra, ACT: AIHW.

[B8] Bainbridge R. Tsey K. McCalman J. Kinchin I. Saunders V. Watkin Lui F.. (2015). No one's discussing the elephant in the room: contemplating questions of research impact and benefit in aboriginal and torres strait islander Australian health research. BMC Pub. Health 15, 696. 10.1186/s12889-015-2052-3 26202429 PMC4511988

[B9] Balabanski A. H. Goldsmith K. Giarola B. Buxton D. Castle S. McBride K.. (2020). Stroke incidence and subtypes in Aboriginal people in remote Australia: a healthcare network population-based study. BMJ Open 10, e039533. 10.1136/bmjopen-2020-039533 33033097 PMC7545633

[B10] Balabanski A. H. Nedkoff L. Brown A. Thrift A. G. Pearson O. Guthridge S.. (2023). Incidence of stroke in the aboriginal and non-aboriginal populations of Australia: a data linkage study. Stroke. 54, 2050–2058. 10.1161/STROKEAHA.122.041975 37325922

[B11] Balabanski A. H. Newbury J. Leyden J. M. Arima H. Anderson C. S. Castle S.. (2018). Excess stroke incidence in young aboriginal people in South Australia: pooled results from two population-based studies. Int. J. Stroke 13, 811–814. 10.1177/1747493018778113 29767602

[B12] Belbasis L. Bellou V. (2018). Introduction to epidemiological studies. Methods Mol. Biol. 1793, 1–6. 10.1007/978-1-4939-7868-7_1 29876887

[B13] Bougie E. (2021). Acute-Care Hospitalizations Among First Nations People, Inuit and Métis: Results from the 2006 and 2011 Canadian Census Health and Environment Cohorts. Ottawa, ON: Statistics Canada. 10.25318/82-003-x202100700002-eng 34288618

[B14] Christensen D. Davis G. Draper G. Mitrou F. McKeown S. Lawrence D.. (2014). Evidence for the use of an algorithm in resolving inconsistent and missing Indigenous status in administrative data collections. Austr. J. Soc. Issues. 49, 423–443. 10.1002/j.1839-4655.2014.tb00322.x

[B15] Commonwealth of Australia (2018). Closing the Gap Prime Minister's Report 2018. Canberra: Department of the Prime Minister and Cabinet.

[B16] Delgado-Rodríguez M. Llorca J. (2004). Bias. JECH 58, 635. 10.1136/jech.2003.008466 PMC1732856 15252064

[B17] Feigin V. Norrving B. Sudlow C. L. M. Sacco R. L. (2018). Updated criteria for population-based stroke and transient ischemic attack incidence studies for the 21st century. Stroke 49, 2248–2255. 10.1161/STROKEAHA.118.022161 30355005

[B18] Feigin V. L. Krishnamurthi R. V. Barker-Collo S. McPherson K. M. Barber P. A. Parag V.. (2015). 30-year trends in stroke rates and outcome in Auckland, New Zealand (1981-2012): a multi-ethnic population-based series of studies. PLoS ONE 10, e0134609. 10.1371/journal.pone.0134609 26291829 PMC4546383

[B19] Huria T. Palmer S. C. Pitama S. Beckert L. Lacey C. Ewen S.. (2019). Consolidated criteria for strengthening reporting of health research involving indigenous peoples: the consider statement. BMC Med. Res. Methodol. 19, 173. 10.1186/s12874-019-0815-8 31399058 PMC6688310

[B20] Katzenellenbogen J. M. Somerford P. Semmens J. B. Codde J. P. (2010). Effect of clearance periods on hospital stroke incidence using linked administrative data. Int. J. Stroke. 5, 336–337. 10.1111/j.1747-4949.2010.00455.x 20636720

[B21] Levine D. A. Duncan P. W. Nguyen-Huynh M. N. Ogedegbe O. G. (2020). Interventions targeting racial/ethnic disparities in stroke prevention and treatment. Stroke 51, 3425–3432. 10.1161/STROKEAHA.120.030427 33104466 PMC7594115

[B22] Leyden J. M. Kleinig T. J. Newbury J. Castle S. Cranefield J. Anderson C. S.. (2013). Adelaide stroke incidence study: declining stroke rates but many preventable cardioembolic strokes. Stroke 44, 1226–1231. 10.1161/STROKEAHA.113.675140 23482602

[B23] National Health and Medical Research Council (2018). Ethical conduct in research with Aboriginal and Torres Strait Islander Peoples and Communities: Guidelines for Researchers and Stakeholders. Canberra: Commonwealth of Australia.

[B24] Office of the National Rural Health Commissioner (2022). Position Statement: Impacts of racism on the health and wellbeing of Indigenous Australians. Canberra: Australian Government.

[B25] Research Data Alliance International Indigenous Data Sovereignty Interest Group (2019). CARE and Principles for Indigenous Data Governance: The Global Indigenous Data Alliance. Available online at: http://www.GIDA-global.org (accessed July 5, 2023).

[B26] Siri S. R. A. Eliassen B. M. Broderstad A. R. Melhus M. Michalsen V. L. Jacobsen B. K.. (2020). Coronary heart disease and stroke in the Sami and non-Sami populations in rural Northern and Mid Norway-the SAMINOR Study. Open Heart 7, 1–5. 10.1136/openhrt-2019-001213 32404487 PMC7228651

[B27] Sjölander P. (2011). What is known about the health and living conditions of the indigenous people of northern Scandinavia, the Sami? Glob. Health Action 4, 8457. 10.3402/gha.v4i0.8457 22007156 PMC3195409

[B28] Stark B. A. Roth G. A. Adebayo O. M. Akbarpour S. Aljunid S. M. Alvis-Guzman N.. (2021). Global, regional, and national burden of stroke and its risk factors, 1990–2019: a systematic analysis for the global burden of disease study 2019. Lancet Neurol. 20, 795–820. 10.1016/S1474-4422(21)00252-0 34487721 PMC8443449

[B29] Sudlow C. L. Warlow C. P. (1996). Comparing stroke incidence worldwide: What makes studies comparable? Stroke 27, 550–558. 10.1161/01.STR.27.3.550 8610328

[B30] Thompson S. C. Woods J. A. Katzenellenbogen J. M. (2012). The quality of indigenous identification in administrative health data in Australia: insights from studies using data linkage. BMC Med. Inform. Decis. Mak. 12, 133. 10.1186/1472-6947-12-133 23157943 PMC3536611

[B31] Trimble B. Morgenstern L. B. (2008). Stroke in minorities. Neurol. Clin. 26, 1177–1190. 10.1016/j.ncl.2008.05.010 19026907 PMC2621018

[B32] United Nations (2007). United Nations Declaration on the Rights of Indigenous, Peoples (UNDRIP): Resolution adopted by the General Assembly on 13 September 2007.

[B33] Youens D. Katzenellenbogen J. Ragavan R. S. Sodhi-Berry N. Carson J. Zemedikun D. Nedkoff L. (2023). Differing definitions of first-ever stroke influence incidence estimates more than trends: a study using linked administrative data. Neuroepidemiology. 10.1159/000534242. [Epub ahead of print]. 37751719

[B34] Zhang Y. Galloway J. M. Welty T. K. Wiebers D. O. Whisnant J. P. Devereux R. B.. (2008). Incidence and risk factors for stroke in American Indians: the strong heart study. Circulation 118, 1577–1584. 10.1161/CIRCULATIONAHA.108.772285 18809797 PMC2754380

